# Phylogeographic patterns of *Lygus pratensis* (Hemiptera: Miridae): Evidence for weak genetic structure and recent expansion in northwest China

**DOI:** 10.1371/journal.pone.0174712

**Published:** 2017-04-03

**Authors:** Li-Juan Zhang, Wan-Zhi Cai, Jun-Yu Luo, Shuai Zhang, Chun-Yi Wang, Li-Min Lv, Xiang-Zhen Zhu, Li Wang, Jin-Jie Cui

**Affiliations:** 1 State Key Laboratory of Cotton Biology, Institute of Cotton Research of CAAS, Anyang, China; 2 Department of Entomology, China Agricultural University, Beijing, China; National Cheng Kung University, TAIWAN

## Abstract

*Lygus pratensis* (L.) is an important cotton pest in China, especially in the northwest region. Nymphs and adults cause serious quality and yield losses. However, the genetic structure and geographic distribution of *L*. *pratensis* is not well known. We analyzed genetic diversity, geographical structure, gene flow, and population dynamics of *L*. *pratensis* in northwest China using mitochondrial and nuclear sequence datasets to study phylogeographical patterns and demographic history. *L*. *pratensis* (n = 286) were collected at sites across an area spanning 2,180,000 km^2^, including the Xinjiang and Gansu-Ningxia regions. Populations in the two regions could be distinguished based on mitochondrial criteria but the overall genetic structure was weak. The nuclear dataset revealed a lack of diagnostic genetic structure across sample areas. Phylogenetic analysis indicated a lack of population level monophyly that may have been caused by incomplete lineage sorting. The Mantel test showed a significant correlation between genetic and geographic distances among the populations based on the mtDNA data. However the nuclear dataset did not show significant correlation. A high level of gene flow among populations was indicated by migration analysis; human activities may have also facilitated insect movement. The availability of irrigation water and ample cotton hosts makes the Xinjiang region well suited for *L*. *pratensis* reproduction. Bayesian skyline plot analysis, star-shaped network, and neutrality tests all indicated that *L*. *pratensis* has experienced recent population expansion. Climatic changes and extensive areas occupied by host plants have led to population expansion of *L*. *pratensis*. In conclusion, the present distribution and phylogeographic pattern of *L*. *pratensis* was influenced by climate, human activities, and availability of plant hosts.

## Introduction

Cytoplasmic and nuclear data combined with coalescent theory are commonly used for phylogeographic studies. They are powerful tools for evaluating the possible influence of climate changes, geological events, environmental changes and hosts on extant population structure and tracing the possible evolutionary history of species [1]. Molecular phylogeographic results have been used to reconstruct the evolutionary history of species by revealing colonization history, range expansion, and spatial and temporal genetic variation [2, 3]. The phylogeographic patterns of many organisms, whose evolution was affected by vicariance [4–6], climatic changes [7], hosts [8] and human interference events [9–11] have been studied using molecular data.

High levels of genetic diversity occur in many species, partially due to proper habitats and sufficient food. However, the level of genetic variation varies widely between species. Compared to the genetic distinctiveness often found in wild montane species, low genetic differentiation of agricultural and public health insect pests is more common (e.g. *Bactrocera dorsalis* [12]; *Aedes albopictus* [13]; *Plutella xylostella* [14]). Weak genetic structure is usually related to habitat changes or to human activities that promote gene flow between populations. In addition, lineage sorting, resulting from short-term coalescence, helps to weaken genetic differentiation [15].

Vicariance is an important factor influencing the geographic pattern of organisms. Vicariance isolates populations, reduces gene flow, and promotes genetic differentiation. As such, it often results in the evolution of distinct genetic lineages. However, when a species possesses high dispersal capacity [9] combined with migration [16], this greatly increases gene flow and reduces the genetic differentiation of populations.

In addition to the role of vicariance, the contribution of climate conditions to phylogeographic structure can be important. *Lygus pratensis* (L.) is a species of ‘continental’ adaptation, drier climate with greater seasonal variation. According to the characteristic biogeographical patterns of continental taxa, we expected that the *Lygus pratensis* population should have more extensive distributions, experience population expansion during parts of the last glaciation [17], relatively high genetic similarity within regional groups of populations, and moderate to high genetic differentiation among populations in currently isolated regions. In northwest China, the climate was typically warm and dry from 1890 to the 1980s. Water has now become the main limiting factor for species survival. In contrast to the 1961 to 1986 period, warm-wet conditions have become more common since 1987. Precipitation and glacial melt water have also increased in the Xinjiang and Hexi Corridor areas [18]. The climate change from warm-dry to warm-wet has increased vegetation diversity. Rainwater and glacial melt water have provided ample irrigation water for agriculture. The phylogeography of many insects has been studied in northwest China including species such as *Sitodiplosis mosellana* (Gehin) [19]. Northwest China has a diverse topography, including deserts, basins, mountains, rivers, and lakes. This diversity, combined with the effects of modern climatic oscillations, has promoted the diversification and divergence of plants and animals.

Large amounts of cotton are cultivated in the Xinjiang region and cotton trade commonly occurs among the planting areas [20]. *Lygus pratensis* can move great distances when transported as nymphs or eggs on host plant material. Passive dispersal of a species via other organisms or hosts occurs in many animals. Eggs of zooplankton can survive passage through the guts of migratory waterbirds and disperse to isolated habitats [21]. Chironomids (Diptera) can migrate great distances when transported as larvae via birds [22].

*Lygus pratensis* (Hemiptera: Miridae) is cosmopolitan and found in Europe, Northern Africa, Middle East, Northern India, China, and Siberia. It is a native species in northwest China. *L*. *pratensis* has many host plants but cotton and alfalfa are most important [23]. Substantial amounts of cotton are produced in the Xinjiang area of northwest China [24], and large areas of alfalfa are cultivated in Gansu and Ningxia. This region is the primary range of *L*. *pratensis*, and it is an important cotton pest. Both nymphs and adults attack various plant parts resulting in significant quality and yield losses. Relatively high humidity (≥75%) and warm temperatures (25°C to 35°C) promote egg and nymph development. *L*. *pratensis* populations often increase after significant rainfall [24]. Populations can be substantially larger along rivers compared with locations away from rivers [25]. *L*. *pratensis* has a high flight capacity and can ascend to 1,000 m above the ground [26]. For an important agriculture pest such as *L*. *pratensis* with high dispersal capability, it is important to clarify its population genetic structure and demographic history. This information may help in developing sustainable management strategies that reduce population sizes and minimize economic losses.

In this study, we sequenced five DNA fragments (*COI*, *COII*, *Cytb*, *ND5*, and 16S rRNA) from mitochondria (mtDNA) and three ribosomal DNA (rDNA) sequences [ITS2 (internal transcribed spacer 2), 5.8S and 28S] from 286 adults individuals, originating from nine populations covering the main distribution region of this species in China. Our aims were to (i) determine the phylogeographic structure and possible influential factors; (ii) study the demographic history and gene flow among populations; and (iii) identify human activities and ecological conditions responsible for the current population structure and distribution patterns.

## Materials and methods

### Ethics statement

Collection of *L*. *pratensis* for this study did not require permits because *L*. *pratensis* is a ubiquitous cotton pest. The field studies did not involve the use of, nor did they interfere with, endangered or protected species.

### Sampling and laboratory procedures

**Fig 1:**
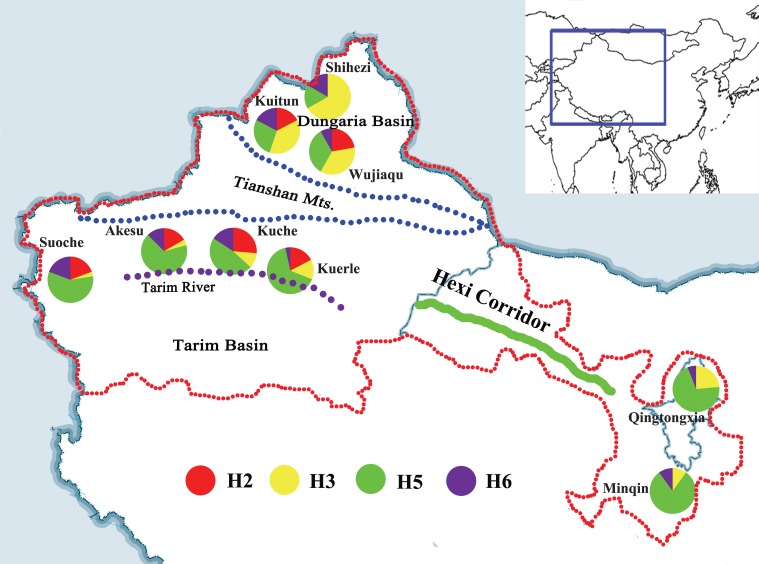
Geographical features and distribution of dominant haplotypes of nine sampled populations of *Lygus pratensis* based on based on mtDNA dataset. For the site names see Table A in S1 File. Red indicates haplotype H2; yellow indicates haplotype H3; green indicates haplotype H5; purple indicates haplotype H6. The red dashed line bound the region which represents sample areas; the blue bound the region which represents Tianshan Mountains; the purple dashed line represents Tarim River; the green solid bold line represents Hexi Corridor.

We analysed 286 *L*. *pratensis* adults from northwest China (Fig 1; Table A in S1 File). Total genomic DNA was extracted from each adult using the DNeasy Blood and Tissue Kit (Qiagen, Hilden, Germany). Abdomens were removed before DNA extraction.

Data for five mitochondrial gene sequences and three ribosomal DNA sequences were obtained by polymerase chain reaction (PCR) amplification. All primers were specifically designed for *L*. *pratensis* (*COI*-F, *COI*-R; *COII*-F, *COII*-R; *Cytb*-F, *Cytb*-R; *ND5*-F, *ND5*-R; 16S-F, 16S-R), except for the ITS2, and the genes of 5.8S and 28S, which were amplified using primer pairs P1 [27] and 28Z [28] (Table B in S1 File). PCR was performed in 25 μL of reaction solution containing 400 μM each dNTP, 1 μL each primer (10 pmol), 5 μL of 10× PCR buffer, 0.25 units Ex Taq DNA polymerase (Takara Bio), and concentration of 10 ng of template DNA. The PCR cycling procedure was: initial denaturation at 94°C for 3 min, followed by 30 cycles at 94°C for 30 s, 54–58°C for 30 s, and 72°C for 1 min and a final extension step at 72°C for 5 min. PCR sequences were obtained using the ABI 3730xl DNA Analyzer at Suzhou Jinweizhi Biological Technology Co. Ltd. (Suzhou, China). All analysed samples were sequenced from both directions to check for consistency. Sequences were edited, checked, and aligned using CHROMAS 2.31 (Technelysium, Helensvale, Australia), ContigExpress (a component of Vector NTI Suite 6.0) and MEGA 6.0 [29]. All sequences have been submitted to GenBank under accession numbers KX447148–KX447175 for 16S rRNA, KX447176–KX447218 for *COI*, KX447219–KX447261 for *COII*, KX447262–KX447288 for *Cytb*, KX447289–KX447360 for ITS2 plus 5.8S and 28S, KX447361–KX447403 for *ND5*.

### Sequence variation and genetic structure

The sequence polymorphisms were assessed by calculating statistical parameters for five combined mitochondrial genes *COI*+*COII*+*ND5*+*Cytb*+16S rRNA and three combined nuclear markers 5.8S+ITS2+28S. For nuclear sequences, allele determination is a major obstacle to phylogeographic usage [30]. Therefore, we used Perl scripts in CVhaplot 2.0 [31] to infer alleles from heterozygous sites. The validity of this approach has been demonstrated [32]. This program can reduce allele inference uncertainty by controlling the internal variability of individual algorithms [33], and reformat the input files for several population genetic-based software packages, including PHASE [34], Haplotyper [35], HaploRec [36], Arlequin-EM [37], GCHap [38], Gerbil [39] and HAPINFERX [40]. Consensus datasets were generated by comparing results from these programs. DNA sequences with uncertain results (probability threshold < 80%) were removed from analysis. The number of polymorphic sites (*S*), haplotype diversity (*Hd*), nucleotide diversity (*π*), average number of nucleotide differences (*K*), and number of haplotypes (*Ht*) were calculated using DnaSP 5.0 [41] or Arlequin 3.5 [42] for the mtDNA dataset.

The genetic structures of populations were delimited using the SAMOVA 1.0 program [43] based on mtDNA and rDNA datasets; *K* values (number of groups) were set from 2 to 8 and each independent simulated annealing processes was performed with 100 replicates. For each pre-specified *K* value, the method searches for the best clustering option which was defined as the highest *F*_*CT*_ value (the level of genetic differentiation among groups). When SAMOVA analysis did not adequately delimit populations, the population structure was defined according to zoogeographic divisions of China (two geographic units: Xinjiang and Gansu-Ningxia region) for subsequent analysis.

The genetic distance *F*_*ST*_ matrix was obtained using Arlequin software, and then converted into a matrix of (*F*_*ST*_)/(1- *F*_*ST*_) to obtain linearized estimates. The linear geographic distance (km) was obtained from Google Earth. The Isolation-by-distance (IBD) model was tested using the genetic distance (*F*_*ST*_)/(1- *F*_*ST*_) matrix and the linear geographic distance (km) across sampling areas, with the Mantel test employing 1,000 randomizations in program IBDWS 3.2 [44]. The mean genetic distances among populations were determined with program MEGA 6.0.

Hidden genetic barriers associated with genetic discontinuities across the sample areas were further analyzed using the maximum difference Monmonier’s algorithm in the Barrier 2.2 software [45]. This program uses a computational geometry approach to provide the locations and the directions of barriers.

To verify whether there is asymmetrical gene flow between populations, MIGRATE 3.6 [46] software was used to estimate the effective number of migrants from each population per generation (*N*_*e*_*m*: *N*_*e*_ is the effective population size; *m* is the immigration rate; i.e. *ΘM*: *Θ* is mutation-scaled population size; *M* is mutation-scaled migration rate). Also the gene flow between groups (in the Xinjiang and Gansu-Ningxia region) was assessed based on genetic data. A Bayesian search strategy was used to calculate parameters *Θ* and *M*. The parameters were set as follows: long-chains = 1, long-inc = 20, long-sample = 1,000,000, burnin = 100,000, heating = YES: 1: {1.0, 1.5, 3.0, 6.0}, heated-swap = YES and replicate = YES: 5. In order to obtain consistent results four runs of MIGRATE analysis were performed. In the first run, *Θ* and *M* were estimated from *F*_*ST*_ values, in the subsequent three runs, the parameters of the previous run were used as starting values for the next run until a convergent result was obtained. The datasets of *Θ*, *M* and *ΘM* present here were from the final run. In addition, gene flows among populations were also tested by the Arlequin program.

### Haplotype phylogeny and network analysis

In order to study the haplotype and allele phylogeny of *L*. *pratensis*, we constructed haplotype and allele networks with mtDNA and rDNA datasets in Network 4.6 [47] based on the median-joining algorithm.

### Historical demography

Demographic history of *L*. *pratensis* was studied using parameters of Tajima’s *D*, Fu’s *F*_*S*_, demographic expansion time (τ1) and spatial expansion times (τ2), which were calculated in Arlequin 3.5 based on two datasets. Tajima’s *D* and Fu’s *F*_*S*_ were used to detect departure from the population equilibrium. For a species, departures from the null model might be caused by many factors. One factor is fluctuation in population size (e.g. population expansion) which results in a large amount of low frequency variants and negative D values. Conversely, processes such as recent population bottlenecks, could result in an excess of intermediate frequency variants and positive D values [48, 49]. Large negative *F*_*S*_ values indicated that the population may have experienced a recent range expansion [50]. The parameters of Fu and Li’s F^*^, Fu and Li’s D^*^ were determined using DnaSP 5.0.

We used the Bayesian skyline plot (BSPs) method implemented in BEAST 1.8 [51] to estimate population expansion times for the mtDNA dataset. The best-fit models were used for separate partitions (i.e. each gene) in a simultaneous analysis of mtDNA. The models of nucleotide substitution were based on the Akaike information criteria, and were selected using jModelTest 2.1 [52]. They were: HKY+I for *COI*; GTR for *COII*, *Cytb*, and 16S rRNA; GTR+I for *ND5*; and GTR+I+G for the three nuclear fragments, respectively. Trace 1.4 [53] was used to ensure that ESS_S_ were greater than 200, with a burn-in of 10%. A relaxed uncorrelated lognormal molecular clock was applied, with a pairwise sequence divergence rate of 0.0115 per site per million years for the *COI* gene [54] was used to infer demographic trends because there is no fossil or geological evidence available for calibration. Markov chain Monte Carlo analyses were run for 100 × 10^7^ generations with sampling every 1,000 generations, and the Yule process model was used.

## Results

### Sequence variation and genetic structure

The alignment of the combined mitochondrial dataset included 4,046 bp (*COI*: 856 bp, *COII*: 740 bp, *ND5*: 570 bp, *Cytb*: 910 bp and 16S rRNA: 970 bp) from 286 individuals. No insertions or deletions were detected among the above five fragments. The nuclear dataset included 1942 bp (5.8S: 368 bp, ITS2: 711 bp, 28S: 863 bp), sequences from 220 individuals that were successfully sequenced. Five sequences were filtered out by CVhaplot 2.0 due to poor quality and 215 sequences were used for analyses.

For the mtDNA dataset, 64 haplotypes (including 56 unique haplotypes) were identified. Xinjiang had 49 unique haplotypes, Gansu-Ningxia had 15 unique haplotypes; three haplotypes were shared by populations from both regions (Table C in S1 File). There were 106 polymorphic sites, of which 79 were singleton variable sites and 27 were parsimony-informative sites. The highest polymorphic values were all from the KC population. The lowest values were all from the MQ population (Table 1). High *Hd* and low *π* in populations may result from rapid population growth of ancestral populations [55]. Compared to the Xinjiang group, relatively low genetic diversity occurred in the Gansu-Ningxia group.

**Table 1:** Genetic diversity values for *Lygus pratensis* populations based on the mtDNA dataset.

population	N	*S*	*Ht*	*K*	*Hd* (SD)	*π* (SD)
AKS	36	27	16	4.6778	0.7952 (0.0666)	0.0012 (0.0006)
KT	38	25	13	4.1935	0.8535 (0.0346)	0.0010 (0.0006)
WJQ	47	27	15	4.5347	0.8381 (0.0322)	0.0011 (0.0006)
SC	35	16	8	3.3950	0.6924 (0.0686)	0.0008 (0.0005)
SHZ	8	7	5	2.0714	0.7857 (0.1508)	0.0005 (0.0004)
KC	27	20	12	4.7692	0.8462 (0.0535)	0.0012 (0.0007)
KEL	34	20	9	3.5972	0.6667 (0.0817)	0.0009 (0.0005)
MQ	39	20	11	1.4980	0.6181 (0.0890)	0.0004 (0.0003)
QTX	22	33	8	3.8701	0.6883 (0.0992)	0.0010 (0.0006)
XJ	225	73	52	4.1357	0.9666 (0.0040)	0.0010 (0.0006)
GN	61	46	15	2.3585	0.8060 (0.0422)	0.0006 (0.0004)
Total	286	106	64	3.8632	0.7845 (0.0210)	0.0010 (0.0005)

Note: N, number of individuals; *S*, number of segregating sites; *Ht*, number of haplotypes; *K*, average number of nucleotide differences; *Hd*, haplotype diversity; π, nucleotide diversity; SD = standard deviation; the population code as follows: AKS = Akesu; KT = Kuitun; WJQ = Wujiaqu; SC = Suoche; SHZ = Shihezi; KC = Kuche; KEL = Kuerle; MQ = Minqin; QTX = Qingtongxia; XJ = Xinjiang; GN = Gansu-Ningxia.

SAMOVA analysis based on the mtDNA dataset showed that *F*_*CT*_ values distinctly declined from *K* = 2 to 4. There was a slight decrease of *F*_*CT*_ values from *K* = 5 to 8. *F*_CT_ reached the highest values (*F*_*CT*_ = 0.076, p < 0.05) when *K* = 2 (S1 Fig). Two groups were delimited. One group included populations from the Gansu-Ningxia region (QTX and MQ) except for the SHZ population. The other group included six Xinjiang populations, AKS, KT, WJQ, SC, KC and KEL. For the nuclear dataset, *F*_*CT*_ values clearly declined from *K* = 2 to 5. At *K* = 2, *F*_*CT*_ had the largest values (*F*_*CT*_ = 0.082, p < 0.001) (S2 Fig), which corresponded to the QTX group, while the other combined group included the remaining eight populations. In conclusion, SAMOVA was unable to clearly define population structure according to sample locations. Because of this, two groups, delimited by the zoogeography of China (i.e. Xinjiang and Gansu-Ningxia) were used in subsequent analyses.

**Fig 2:**
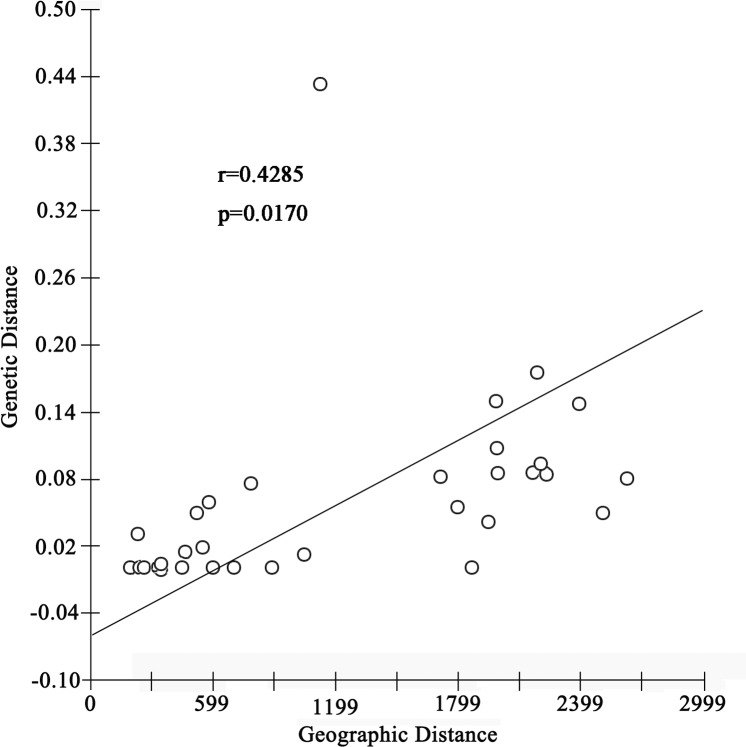
Correlation of genetic distance and geographical distance based on the mtDNA dataset.

**Fig 3:**
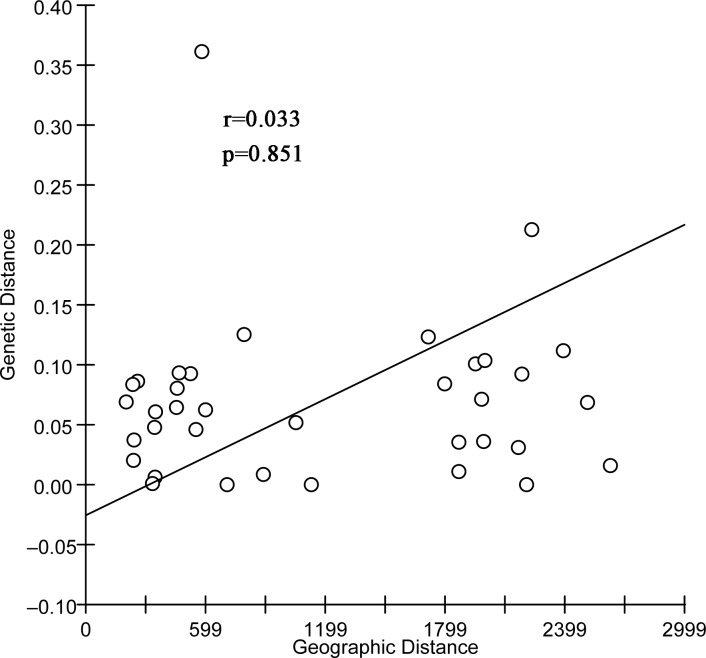
Correlation of genetic distance and geographical distance based on the rDNA dataset.

The IBD test showed significant correlation between genetic differentiation (*F*_*ST*_)/(1*−F*_*ST*_) and geographical distance among populations based on the mtDNA dataset (r = 0.429, p = 0.017, Fig 2). For the nuclear dataset, the hypothesis of a significant correlation was rejected (r = 0.033, p = 0.851) (Fig 3).

Analyses of genetic discontinuities showed a clear division between the Xingjiang and Gansu-Ningxia populations based on mitochondrial (bold red line ‘a’ in S3 Fig) and nuclear (S4 Fig) data. Both datasets supported the existence of a genetic barrier separating the two regions. However, there were no obvious barriers blocking gene flow between the two groups. Therefore, we speculated that geographic isolation might influence the gene flow and this was consistent with the IBD test based on mtDNA dataset.

For the mtDNA dataset, the genetic differentiation among all populations was low but significant (*F*_*ST*_ = 0.0318, p < 0.01). Similar results were found between Xinjiang and Gansu-Ningxia groups (*F*_*CT*_ = 0.07278, p < 0.001). The relatively low pairwise genetic differentiation and only reached a low level of differentiation was also found between populations (Table 2) based on the criteria of genetic differentiation used by Wright (1978) [56]. Differentiation among populations was determined using *F*-statistics where *F*_*ST*_ > 0.25 indicates significant genetic differentiation, 0.25 > *F*_*ST*_ > 0.15 indicates moderate genetic differentiation, 0.15 > *F*_*ST*_ > 0.05 indicates small genetic differentiation, and *F*_*ST*_ < 0.05 indicates negligible genetic differentiation. Nuclear sequences yielded an *F*_*ST*_ = 0.04838 (p < 0.001) for all populations (Table D in S1 File) and group structure was presented in this dataset (*F*_*CT*_ = 0.0296, p < 0.001). Overall, genetic differentiation among populations was low and both defined groups showed weak genetic structure. The mean genetic distance was 0.001 among populations based on the MEGA program.

**Table 2:** Pairwise *F*_*ST*_ values of nine *Lygus pratensis* populations based on the mtDNA dataset.

Population	AKS	KT	WJQ	SC	SHZ	KC	KEL	MQ	QTX
AKS		551.8	708.0	345.0	793.5	259.9	454.5	2393.0	2234.7
KT	0.018		239.1	890.0	241.5	349.4	347.3	2208.6	1952.6
WJQ	0.000	-0.006		1052.9	235.7	457.7	334.5	1983.6	1716.2
SC	0.000	-0.001	0.012		1130.2	600.0	1869.1	2628.6	2513.8
SHZ	0.071	-0.022	0.030	0.041		581.5	525.3	2167.9	1869.1
KC	0.071	-0.002	-0.021	-0.004	0.055		202.8	2185.3	2000.1
KEL	-0.013	0.004	-0.005	-0.002	0.047	-0.015		1993.2	1798.4
MQ	0.128***	0.085**	0.130***	0.074**	0.079	0.149***	0.097**		468.1
QTX	0.078**	0.040*	0.076*	0.046	-0.010	0.078*	0.051*	0.015	

*p < 0.05

**p < 0.02

***p < 0.001

the values above diagonal indicate geographic distance; the values below diagonal indicate Pairwise *F*_*ST*_.

BI-based analysis using MIGRATE 3.6 showed that all populations are well connected in both directions, with high levels of gene flow based on both datasets (Table 3; Table E in S1 File). Contrary to the expectation that long-term gene flow between populations tends to be asymmetrical, the gene flow of this species was symmetrical between each pair of the nine populations. For the mtDNA dataset, asymmetrical effective migrants per generation (*N*_*e*_*m*) were observed between populations, and for the Akesu and Suoche populations, the levels were relatively high (Table F in S1 File). More individuals immigrate into the Akesu population from other populations; conversely, large numbers of individuals tend to emigrate from the Suoche population. In five populations (Kuitun, Wujiaqu, Suoche, Kuerle and Minqin), the mean values for numbers of immigrants were very low (*N*_*e*_*m* < 2); the remaining four populations (Qingtongxia, Akesu, Kuche and Shihezi) had high numbers of immigrants (*N*_*e*_*m*: 5.79–15.86). When the geographical groups (Xinjiang and Gansu-Ningxia) were analysed, the effective population number from Xinjiang (*θ* = 0.006) was relatively higher than Gansu-Ningxia (*θ* = 0.002).

**Table 3:** Migration parameter estimates (mean *M* and *θ* values) based on the mtDNA dataset for nine populations of *Lygus pratensis*.

Population		AKS	KT	WJQ	SC	SHZ	KC	KEL	MQ	QTX
*i*	*θi*	→*i*	→*i*	→*i*	→*i*	→*i*	→*i*	→*i*	→*i*	→*i*
AKS	0.01599		583.4	599.1	742.1	601.0	594.5	658.6	646.2	514.1
KT	0.00256	529.5		605.1	686.8	660.3	570.7	575.0	563.9	485.0
WJQ	0.00157	501.2	531.2		662.9	634.0	524.8	562.1	528.8	462.2
SC	0.00038	440.5	512.1	528.0		562.8	487.4	507.5	490.4	428.9
SHZ	0.01203	480.9	529.6	570.8	585.2		498.0	513.9	514.8	504.0
KC	0.01317	555.8	557.6	621.7	711.7	628.8		623.6	581.9	488.4
KEL	0.00091	561.3	496.9	554.9	629.1	596.8	516.2		539.0	505.6
MQ	0.00093	510.6	496.0	488.6	591.4	555.8	456.9	502.5		479.7
QTX	0.02193	540.1	552.3	582.3	642.8	636.8	539.3	588.2	723.2	

Note: The ‘***i***’ indicates the population code in the first column; ‘***θ***’ indicates the mutation-scaled population size; ‘*M*’ indicates mutation-scaled migration rate.

Compared to the mtDNA, the rDNA dataset showed that the mean values of *N*_*e*_*m* among populations were high except for the Akesu and Kuerle populations. Asymmetrical effective migrants per generation (*N*_*e*_*m*) were observed among Akesu and the other seven populations, with more individuals emigrating from Akesu to the other regions. Similar asymmetrical migration occurred between Kuerle and the other seven populations (Table G in S1 File). This is similar to the results of the mtDNA that showed that the effective population number of Xinjiang was relatively higher than Gansu-Ningxia.

The results based on combined rDNA and mtDNA datasets (eight molecular markers were cascaded, the length of 5988bp, 183 sequences were used) were similar to the above two datasets, high level of gene flow were found among populations (Table H in S1 File), asymmetrical *N*_*e*_*m* were found more frequently between Shihezi and each of these three populations (Kuitun, Kuerle and Qingtongxia), and between Kuerle and each of these three populations (Shihezi, Kuche and Minqin) (Table I in S1 File). The relatively higher *N*_*e*_*m* values were also found in Xinjiang region.

A high level of gene flow was also demonstrated using the Arlequin program analysis based on the mtDNA and rDNA datasets. A large amount of gene flow values *Nm* > 1 (Table 4), indicate that gene exchange among populations was frequent. This is consistent with the Wright (1931) proposal that if the *Nm* (gene flow) > 1, a population will be no significant genetic differentiation and it is replaced by immigrants [57]. The *Nm* ranged from 1.17 to 288.59 based on the rDNA dataset. The mtDNA dataset showed *Nm* values ranging from 1.70 to 948.97

**Table 4:** Gene flow among nine populations based on the rDNA dataset.

	AKS	KT	WJQ	SC	SHZ	KC	KEL	MQ	QTX
AKS									
KT	5.436								
WJQ	inf	12.293							
SC	5.244	29.552	4.825						
SHZ	1.997	6.723	2.991	inf					
KC	2.902	4.110	3.109	4.006	6.921				
KEL	3.885	40.588	288.586	7.058	2.700	3.622			
MQ	2.239	inf	3.505	15.751	8.069	2.712	6.943		
QTX	1.175	2.481	2.030	3.647	22.854	2.414	2.973	2.683	

Note: The ‘inf’ indicates infinite.

### Phylogenies and networks

**Fig 4:**
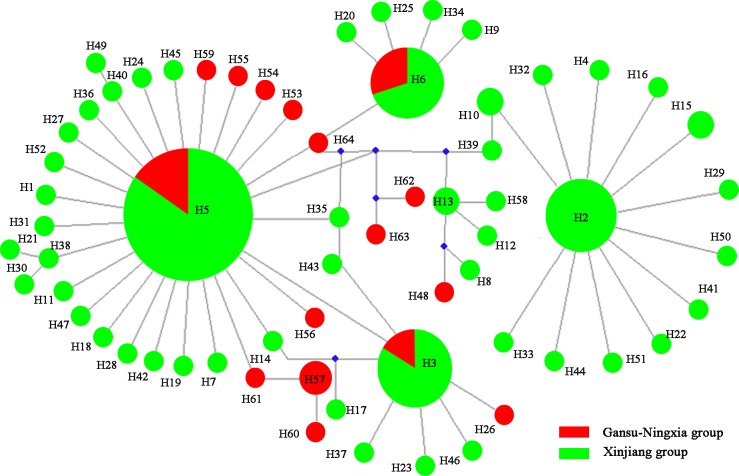
Haplotype network of *Lygus pratensis* based on the mtDNA dataset. Haplotype circle size indicates the number of individuals observed. Colours correspond to different regions. The red indicates Gansu-Ningxia populations; the green indicates Xinjiang populations.

Network analysis for the mitochondrial dataset revealed that H2, H3, H5 and H6 haplotypes were the four most frequent haplotypes, which included 12%, 15%, 42% and 9% individuals, respectively (Fig 4). H3, H5 and H6 occurred in all of the study areas. H2 was a unique haplotype from the Xinjiang region. The haplotype network displayed a star-like pattern, with the four common haplotypes in the center.

**Fig 5:**
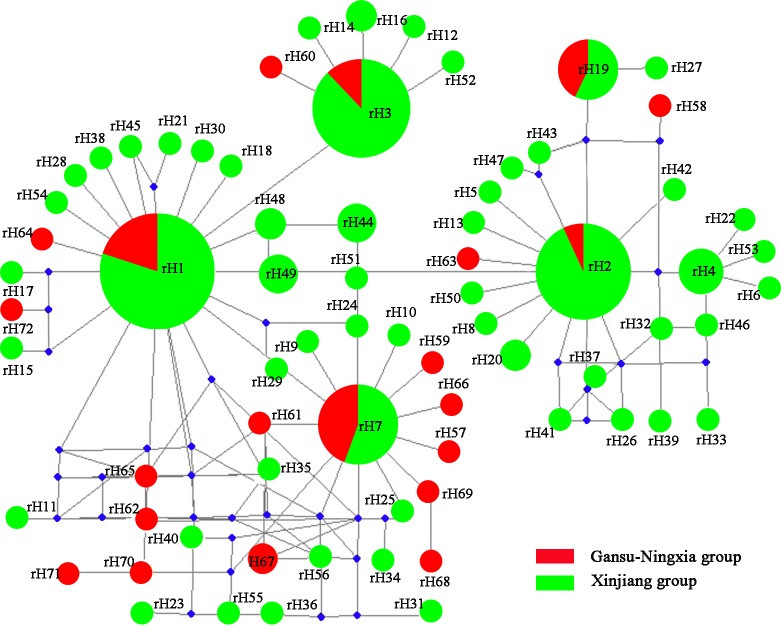
Allele network of *Lygus pratensis* based on the rDNA dataset. Allele circle size indicates the number of individuals observed. Blue solid diamond represents missing intermediate haplotypes. Colours correspond to different regions. The red indicates Gansu-Ningxia populations; the green indicates Xinjiang populations. Blue solid diamond represents missing intermediate alleles.

For the nuclear dataset, rH1, rH2, rH3, and rH7 were major alleles, which accounted for 23%, 13%, 15%, and 8% all individuals, respectively. The four main alleles were all shared by the Xinjiang and Gansu-Ningxia populations. All of these were in the star center (Fig 5). This is consistent with the population structure analyses, in which of no obvious structure was present.

### Demographic dynamics

In the neutrality test, the significance of the variation depends on the statistical test and sampling region. For the mtDNA and rDNA datasets, Tajima’s *D*, Fu’s *Fs*, Fu and Li’s D^*^ and Fu and Li’s F^*^ were highly significant whether the nine populations were considered as one unit or they were delimited into the Xinjiang and Gansu-Ningxia groups (Table 5).

**Table 5:** Demographic analysis of *Lygus pratensis* based on the mtDNA and rDNA datasets.

Dataset	Group	Tajima’*D*	Fu’s *Fs*	Fu’s and Li’s F*	Fu and Li’s D*	τ1	τ2
mtDNA dataset	Xinjiang	-2.00151***	-25.50833***	-6.78897**	-8.77147**	10.010	7.135
	Gansu-Ningxia	-2.54324***	-9.84987***	-4.89895**	-5.03533**	1.287	1.634
	Total	-2.33482**	-54.894***	-7.72194**	-10.35008**		
rDNA dataset	Xinjiang	-2.51048***	-26.44548***	-6.38119**	-7.62277**	1.131	1.135
	Gansu-Ningxia	-2.32924***	-16.09288***	-4.08833**	-4.05526**	1.141	1.141
	Total	-2.61951***	-93.014***	-7.08043**	-8.89083**		

*p < 0.05

**p < 0.02

***p < 0.001

τ1, demographic expansion time; τ2, spatial expansion time

For the mtNDA dataset, the test results indicated that overall population expansion times were relatively short, and that the Xinjiang populations have longer population expansion times than the Gansu-Ningxia populations (Table 5). However, the nuclear dataset showed that expansion occurred at similar times for Xinjiang and Gansu-Ningxia populations.

**Fig 6:**
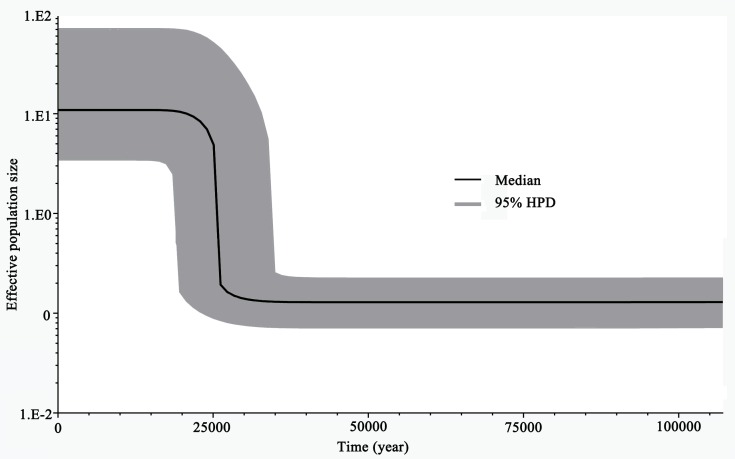
Historical demographic analysis of *Lygus pratensis* based on a Bayesian skyline plot using the mtDNA dataset. The X-axis represents years before present, and the Y-axis represents the estimated effective population size. The solid curve represents median effective population size; the shaded range represents 95% HPD limits.

BSPs analysis, based on the mtDNA dataset, revealed clear evidence of recent population expansion. The effective population size of all the populations remained stable for a long period, then showed a sudden phase of population growth (beginning at about 25,000 years before present), which lasted until recently (Fig 6). Time to the most recent common ancestor (TMRCA) of the Xinjiang clade ranged from 31,500 to 75,100 years before present, and TMRCA of the Gansu-Ningxia clade ranged from 31,400 to 74,900 years before present. TMRCA for both major clades occurred during the Late Pleistocene period (9,700−126,000±5,000) [58].

## Discussion

Studies on the relationship between population genetic structure and host plants of agricultural insect pests are difficult and complicated, especially for species that have diverse host plants and high dispersal capacity. Our previous study of the population dynamics of *L*. *pratensis* on different host plants in northwest China, showed that this species mainly feeds on cotton and alfalfa. However, population size is obviously reduced in groups feeding on native host plants (e.g. *Sophora alopecuroides* Linn, *Artemisia frigida* Willd, *Alhagi sparsifolia* Shap). Therefore, we did not examine molecular markers on samples from native host plants of *L*. *pratensis*. We only collected samples from cultivated cotton and alfalfa. Native host plants might play a role in the population genetics of *L*. *pratensis*, but this possibility was not discussed here because no insect samples from these hosts were available.

### Genetic diversity among populations and groups

Compared to other mirid species [3, 5], *L*. *pratensis* has relatively high genetic diversity based on the mtDNA and rDNA datasets. The mtDNA dataset indicated relatively high genetic diversity in the Xinjiang region but low genetic diversity in Gansu-Ningxia populations. However, the rDNA dataset indicated similar genetic diversity in both groups. Discrepancies between rDNA and mtDNA genetic markers could be due to the maternal inheritance of mitochondrial DNA; and the ITS rDNA regions evolving more rapidly than the coding regions. These regions would therefore show more polymorphisms which would be useful for phylogeographical studies. We suggest that the relatively high genetic diversity in the Xinjiang region based on the mtDNA dataset is related to the host plants. The abundance of cotton hosts provides ample food for *L*. *pratensis*, resulting in successful reproduction, high rates of population growth, and large populations. Neutrality tests also indicated that the Xinjiang populations have experienced recent expansion. These results demonstrate that larger populations tend to possess high genetic diversity compared to smaller populations [15].

Based on the mtDNA dataset of the Gansu-Ningxia populations, an indication of colonization was found in the genetic diversity patterns showing lower genetic diversity (Table 3), and a higher percentage of derived haplotypes [59]. Low genetic diversity can be attributed to absence of the high frequency haplotype H2, which may have drifted in the Gansu-Ningxia populations due to their smaller population sizes. In addition, few founder individuals (low *Θ* value) and recent population expansion (Table 4) indicated that the Gansu-Ningxia populations may experience genetic drift and loss of genetic diversity in the process of colonization. This may also be a consequence of sampling. We have only collected two populations from this region in view of the small geographical area of Gansu-Ningxia. If more Gansu-Ningxia populations are tested and shown to be similar to those reported here, this would support our conclusions.

### Phylogeographic structure of *L*. *pratensis*

The geographical division between Xinjiang and Gansu-Ningxia was studied using mtDNA analyses. The overall genetic structure was weak; and nuclear DNA did not show the expected genetic pattern. Instead, nuclear DNA showed a lack of distinct genetic structure across the entire sample region. *L*. *pratensis* is a continental species, and it is suggest that lack of monophyly at the population level may result from incomplete lineage sorting. This can occur when populations have recently diverged, but retaining unique ancestral polymorphisms [15]. We speculate that the former range of *L*. *pratensis* may have been continuous and then, in relatively recent times, (Holocene) it was divided. The time to the most recent common ancestor of both groups occurred in MIS3 periods, and the amounts of shared haplotype/alleles among current populations all support this conclusion. In addition, the significant IBD patterns were found based on the mtDNA dataset. This pattern usually results from limited gene flow and increased genetic differentiation among geographical habitats. However, high levels of gene flow and low genetic differentiation were found based on the mtDNA dataset in *L*. *pratensis* from our sample regions.

Our original hypothesis was that the recent divergence among *L*. *pratensis* populations is related to host plant introduction events. For example, cotton and alfalfa were introduced into China relatively recently. Asiatic cotton from Southeast Asia was introduced to China in 221 B.C. (during the Qin and Han dynasties) and cotton was intensively cultivated from 1840 to 1921 (i.e. the end of the Qing Dynasty) [60]; Alfalfa was introduced to China in 115 B.C. (Han Dynasty period) [61]. During the sampling of *L*. *pratensis*, we found that cotton is one of the most important hosts in the Xinjiang region. However, in the Gansu-Ningxia region, alfalfa is the most important host. We suggest that the short time period of differentiation on alternate hosts might contribute to the weak genetic structure. The genetic data support this hypothesis; the most common haplotype H5 was predominant over other haplotypes (S3 Table), and this occurred in all populations. In contrast, haplotype H2 was exclusive to the Xinjiang populations and a large number of unique haplotypes (30) were found in these populations. The biased distribution of haplotypes among populations and reduced divergence indicated lineage sorting of the five mitochondrial loci in populations. In addition, introgression may disturb the reciprocal monophyly of populations and should occur when there is a high level of gene flow.

Secondly, the genetic structure of *L*. *pratensis* may result from geography exerting different selective pressures within each region. The complex landscape of Xinjiang, including the Tian Shan Mountains, Dzungarian Basin (with the Gurbantunggut Desert), and Tibetan Basin (with the Taklamakan Desert), present diverse habitat climate types for *L*. *pratensis*. The Tian Shan Mountains may have had an important effect on the current species distribution and this has been demonstrated in previous studies [62]. However, gene flow between the northern and southern Xinjiang populations of *L*. *pratensis* was not prevented by the Tian Shan Mountains. Therefore, we suggest that the homogeneity of populations was more likely associated with the high dispersal ability of *L*. *pratensis* and the effects of human activities.

Long distance dispersal of insects by human transport or host dissemination has been documented in *Nesidiocoris tenuis* [3], *Grapholita molesta* [63], and *Dermacentor variabilis* [64]. The Hexi Corridor connects the Gansu-Ningxia and Xinjiang regions, and is located within our sampling range of *L*. *pratensis*. In ancient China (since 1 B.C; i.e. the Xihan Dynasty), the Hexi Corridor was an important corridor for traffic between inland China and the Western Region; it is now among the most centralized areas of the economy related to transportation, communication, education, and information [65, 66]; as well as major areas for western development. Cotton is a key economic crop that has travelled through the Xinjiang and Hexi Corridor regions, and finally reached Central China [60]. Alfalfa is an important animal food that was introduced to China from Iran. The frequent trade associated with these two host plants could have increased the chance of passive dispersal of *L*. *pratensis* among populations in western China and increased the level of gene flow.

### Factors affecting population expansion of *L*. *pratensis*

The Neutrality test showed significant negative parameters. A star-shaped network and BSPs analysis indicted a period of rapid population growth. These results indicate recent population expansion in *L*. *pratensis*. However, the major factors affecting the population expansion of *L*. *pratensis* remain unclear. We propose that humidity, precipitation, and host plant availability might be the main factors limiting population expansion. Du (1996) found that air temperature has increased in winter and precipitation has increased in the summers since 1977 in the western arid region of China [67]. In addition, large amounts of water from snow and glacial melting have increased the drainage amount of rivers [68]. These studies indicate that the climate in the western arid region of China is changing, favouring the growth of more diverse vegetation and providing alternate hosts for *L*. *pratensis*. *L*. *pratensis* has a period of rapid population growth from June through September and increasing rainfall in the summer can benefit population growth. In conclusion, increased precipitation and availability of numerous host plants might have resulted in *L*. *pratensis* population expansion.

## Supporting information

S1 Fig Fixation indices corresponding to the number of groups (K) inferred by SAMOVA analysis based on mtDNA dataset. (TIF)

S2 Fig Fixation indices corresponding to the number of groups (K) inferred by SAMOVA analysis based on the rDNA dataset. (TIF)

S3 Fig Predicted locations of barriers based on the Barrier program and the mtDNA dataset. The genetic barriers are shown in red line ‘a’.
(TIF)

S4 Fig Predicted locations of barriers based on the Barrier program and the rDNA dataset. The genetic barriers are shown by red line ‘a’.
(TIF)

S1 File A supplemental file containing nine tables. Collection information of *Lygus pratensis* from each site (Table A). Primer sequences used for amplification in this study (Table B). Haplotype frequency by population based on the mtDNA dataset of *Lygus pratensis* (Table C). Pairwise *F*_*ST*_ values of nine populations of *Lygus pratensis* based on the rDNA dataset (Table D). Migration parameter (mean *M* and *θ* values) estimates for nine populations of *Lygus pratensis* based on the rDNA dataset (Table E). Number of effective migrants per generation (*Nem*) for nine populations of *Lygus pratensis* based on the mtDNA dataset (Table F). Number of effective migrants per generation (*Nem*) for nine populations of *Lygus pratensis* based on the rDNA dataset (Table G). Migration parameter (mean *M* and *θ* values) estimates for nine populations of *Lygus pratensis* based on the combined mtDNA and rDNA datasets (Table H). Number of effective migrants per generation (*Nem*) for nine populations of *Lygus pratensis* based on the combined mtDNA and rDNA datasets (Table I).
(DOCX)
