# Supplementary figures and images for: Phylogeographic patterns of Lygus pratensis (Hemiptera: Miridae): Evidence for weak genetic structure and recent expansion in northwest China
Source: PLoS One. 2017 Apr 3;12(4):e0174712. doi: 10.1371/journal.pone.0174712 (PMC5378377; doi:10.1371/journal.pone.0174712)

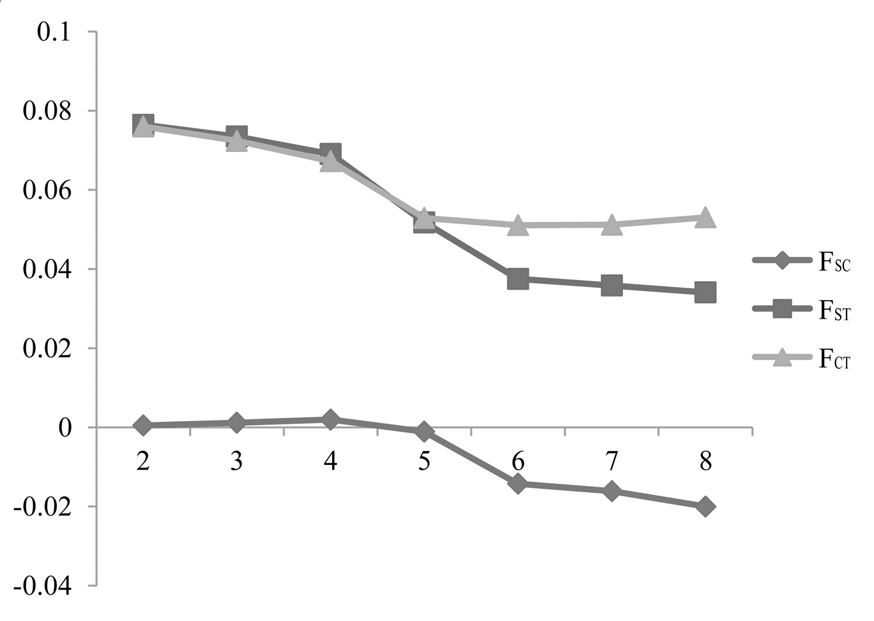

Supplement: S1 Fig — (TIF) [file pone.0174712.s001.tif]

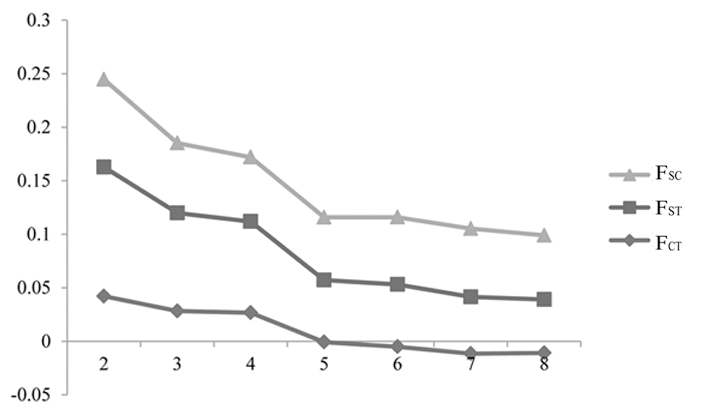

Supplement: S2 Fig — (TIF) [file pone.0174712.s002.tif]

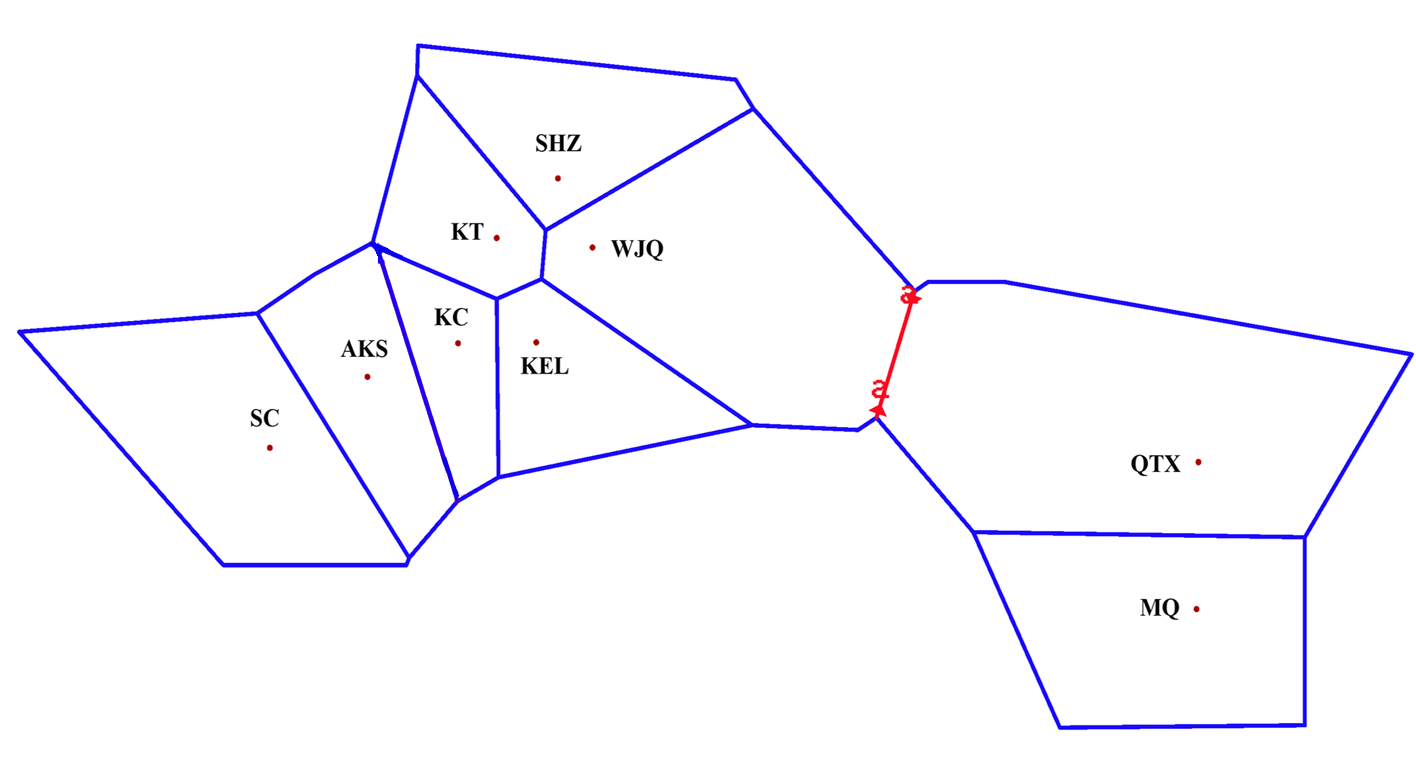

Supplement: S3 Fig — The genetic barriers are shown in red line ‘a’. (TIF) [file pone.0174712.s003.tif]

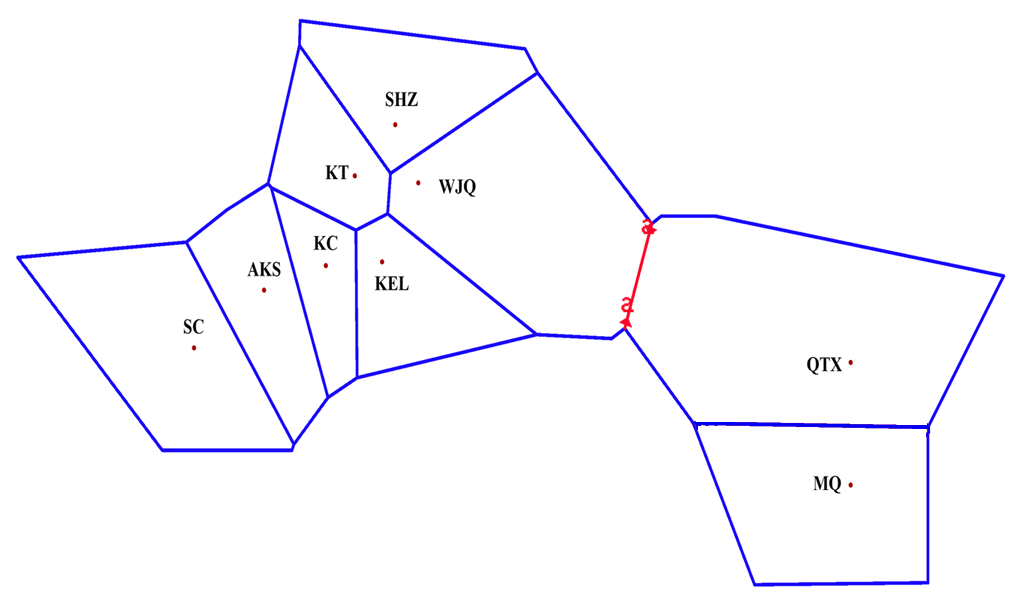

Supplement: S4 Fig — The genetic barriers are shown by red line ‘a’. (TIF) [file pone.0174712.s004.tif]
