# Supplementary material for: Phylogeographic patterns of Lygus pratensis (Hemiptera: Miridae): Evidence for weak genetic structure and recent expansion in northwest China
Source: PLoS One. 2017 Apr 3;12(4):e0174712. doi: 10.1371/journal.pone.0174712 (PMC5378377; doi:10.1371/journal.pone.0174712)
Supplement: S1 File — Collection information of Lygus pratensis from each site (Table A). Primer sequences used for amplification in this study (Table B). Haplotype frequency by population based on the mtDNA dataset of Lygus pratensis (Table C). Pairwise FST values of nine populations of Lygus pratensis based on the rDNA dataset (Table D). Migration parameter (mean M and θ values) estimates for nine populations of Lygus pratensis based on the rDNA dataset (Table E). Number of effective migrants per generation (Nem) for nine populations of Lygus pratensis based on the mtDNA dataset (Table F). Number of effective migrants per generation (Nem) for nine populations of Lygus pratensis based on the rDNA dataset (Table G). Migration parameter (mean M and θ values) estimates for nine populations of Lygus pratensis based on the combined mtDNA and rDNA datasets (Table H). Number of effective migrants per generation (Nem) for nine populations of Lygus pratensis based on the combined mtDNA and rDNA datasets (Table I). (DOCX) [file pone.0174712.s005.docx]

**PLoS one**

**SUPPORTING INFORMATION**

**Phylogeographic patterns of *Lygus pratensis* (Hemiptera: Miridae): evidence of weak genetic structure and** **recent expansion in northwest China**

Li-Juan Zhang^1^, Wan-Zhi Cai^2^, Jun-Yu Luo^1^, Shuai Zhang^1^, Chun-Yi Wang^1^, Li-Min Lv^1^, Xiang-Zhen Zhu^1^, Li Wang^1^, Jin-Jie Cui^1*^

^1^ State Key Laboratory of Cotton Biology, Institute of Cotton Research of CAAS, Anyang, 455000, China

^2^ Department of Entomology, China Agricultural University, Beijing 100193, China

^*^Author for correspondence:

E-mail: aycuijinjie@163.com (JJ C)

**Table A. Collection information of *Lygus pratensis* from each site.**

| **Population** | **Detailed locality**  **information** | **Sample size** | **Lat. (°N)** | **Long. (°E)** | **Date** |
| --- | --- | --- | --- | --- | --- |
| Xinjiang |  |  |  |  |  |
| AKS | Akesu, Xinjiang | 41 | 40°27'48'' | 80°19'13'' | VII. 2015 |
| KT | Kuitun, Xinjiang | 42 | 44°26'42'' | 84°7'25'' | VIII. 2015 |
| WJQ | Wujiaqu, Xinjiang | 62 | 44°10'25'' | 87°18'21'' | VIII. 2015 |
| SC | Suoche, Xinjiang | 41 | 38°25'33'' | 77°17'38'' | VII. 2015 |
| SHZ | Shihezi, Xinjiang | 20 | 46°10'31'' | 86°2'5'' | VII. 2015 |
| KC | Kuche, Xinjiang | 37 | 83°8'1'' | 41°25'30'' | VIII. 2015 |
| KEL | Kuerle, Xinjiang | 43 | 41°27'4'' | 85°33'36'' | VII. 2015 |
| Gansu-Ningxia |  |  |  |  |  |
| MQ | Minqin, Gansu | 44 | 33°48'10'' | 106°2'54'' | VIII. 2015 |
| QTX | Qingtongxia, Ningxia | 43 | 38°1'7'' | 106°6'29'' | VII. 2015 |

**Table B. Primer sequences used for amplification in this study.**

| **Gene** | **Name** | **Sequence (5'–3')** |
| --- | --- | --- |
| *COI* | COI-F | GAATTAGGAATACCCGGATCA |
|  | COI-R | CTCCAGTAAGACCTCCTATTGTAAA |
| *COII* | COII-F | TGGCAGAATAAGTGCCATGA |
|  | COII-R | GAGACCAATGCTTTCTTTCAGC |
| *Cytb* | Cytb -F | TCTAATTGATCTTCCTAGCCCAAG |
|  | Cytb -R | CCGTGCTCCAATTCATGTTA |
| *ND5* | ND5-F | AAACATAATTACCTGAACCCATGAA |
|  | ND5-R | TCTTCAACTTTAGTAACTGCAGGAG |
| 16S rRNA | 16S-F | TTTGGGCTTACCTTTTGTATCA |
|  | 16S-R | AAAAATCTGGTCCTTTCGTACT |
| 5.8S+ITS2+28S | 28Z | AGACTCCTTGGTCCGTGTTTC |
|  | P1 | ATCACTCGGCTCGTGGATCG |

**Table C. Haplotype frequency by population based on the mtDNA dataset of *Lygus pratensis*.**

|  | **AKS** | **KT** | **WJQ** | **SC** | **SHZ** | **KC** | **KEL** | **MQ** | **QTX** |
| --- | --- | --- | --- | --- | --- | --- | --- | --- | --- |
| H1 | 1 |  |  |  |  |  |  |  |  |
| H2 | 4 | 5 | 8 | 6 |  | 5 | 5 |  |  |
| H3 | 1 | 11 | 13 | 1 | 4 | 2 | 4 | 3 | 4 |
| H4 | 1 |  |  |  |  |  |  |  |  |
| H5 | 16 | 8 | 12 | 18 | 1 | 9 | 19 | 24 | 12 |
| H6 | 3 | 5 | 3 | 6 | 1 | 3 | 1 | 3 | 1 |
| H7 | 1 |  |  |  |  |  |  |  |  |
| H8 | 1 |  |  |  |  |  |  |  |  |
| H9 | 1 |  |  |  |  |  |  |  |  |
| H10 | 1 | 1 |  |  |  |  |  |  |  |
| H11 | 1 |  |  |  |  |  |  |  |  |
| H12 | 1 |  |  |  |  |  |  |  |  |
| H13 | 1 |  |  |  |  |  | 1 |  |  |
| H14 | 1 |  |  |  |  |  |  |  |  |
| H15 | 1 |  |  |  |  | 1 |  |  |  |
| H16 | 1 |  |  |  |  |  |  |  |  |
| H17 |  | 1 |  |  |  |  |  |  |  |
| H18 |  | 1 |  |  |  |  |  |  |  |
| H19 |  | 1 |  |  |  |  |  |  |  |
| H20 |  | 1 |  |  |  |  |  |  |  |
| H21 |  | 1 |  |  |  |  |  |  |  |
| H22 |  | 1 |  |  |  |  |  |  |  |
| H23 |  | 1 |  |  |  |  |  |  |  |
| H24 |  | 1 |  |  |  |  |  |  |  |
| H25 |  |  | 1 |  |  |  |  |  |  |
| H26 |  |  | 1 |  |  |  |  |  |  |
| H27 |  |  | 1 |  |  |  |  |  |  |
| H28 |  |  | 1 |  |  |  |  |  |  |
| H29 |  |  | 1 |  |  |  |  |  |  |
| H30 |  |  | 1 |  |  |  |  |  |  |
| H31 |  |  | 1 |  |  |  |  |  |  |
| H32 |  |  | 1 |  |  |  |  |  |  |
| H33 |  |  | 1 |  |  |  |  |  |  |
| H34 |  |  |  | 1 |  |  |  |  |  |
| H35 |  |  |  | 1 |  |  |  |  |  |
| H36 |  |  |  | 1 |  |  |  |  |  |
| H37 |  |  |  | 1 |  |  |  |  |  |
| H38 |  |  |  |  | 1 |  |  |  |  |
| H39 |  |  |  |  | 1 |  |  |  |  |
| H40 |  |  |  |  |  | 1 |  |  |  |
| H41 |  |  |  |  |  | 1 |  |  |  |
| H42 |  |  |  |  |  | 1 |  |  |  |
| H43 |  |  |  |  |  | 1 |  |  |  |
| H44 |  |  |  |  |  | 1 |  |  |  |
| H45 |  |  |  |  |  | 1 |  |  |  |
| H46 |  |  |  |  |  | 1 |  |  |  |
| H47 |  |  |  |  |  |  | 1 |  |  |
| H48 |  |  |  |  |  |  | 1 |  |  |
| H49 |  |  |  |  |  |  | 1 |  |  |
| H50 |  |  |  |  |  |  | 1 |  |  |
| H51 |  |  | 1 |  |  |  |  |  |  |
| H52 |  |  | 1 |  |  |  |  |  |  |
| H53 |  |  |  |  |  |  |  | 1 |  |
| H54 |  |  |  |  |  |  |  | 1 |  |
| H55 |  |  |  |  |  |  |  | 1 |  |
| H56 |  |  |  |  |  |  |  | 1 |  |
| H57 |  |  |  |  |  |  |  | 2 | 1 |
| H58 |  |  |  |  |  |  |  | 1 |  |
| H59 |  |  |  |  |  |  |  | 1 |  |
| H60 |  |  |  |  |  |  |  | 1 |  |
| H61 |  |  |  |  |  |  |  |  | 1 |
| H62 |  |  |  |  |  |  |  |  | 1 |
| H63 |  |  |  |  |  |  |  |  | 1 |
| H64 |  |  |  |  |  |  |  |  | 1 |

**Table D. Pairwise *F_ST_* values of nine populations of *Lygus pratensis* based on the rDNA dataset.**

| **Population** | **AKS** | **KT** | **WJQ** | **SC** | **SHZ** | **KC** | **KEL** | **MQ** | **QTX** |
| --- | --- | --- | --- | --- | --- | --- | --- | --- | --- |
| AKS |  |  |  |  |  |  |  |  |  |
| KT | 0.044 |  |  |  |  |  |  |  |  |
| WJQ | -0.001 | 0.020 |  |  |  |  |  |  |  |
| SC | 0.046** | 0.008 | 0.049** |  |  |  |  |  |  |
| SHZ | 0.111 | 0.036 | 0.077 | -0.013 |  |  |  |  |  |
| KC | 0.079** | 0.057** | 0.074*** | 0.059** | 0.035 |  |  |  |  |
| KEL | 0.060* | 0.006 | 0.001 | 0.034* | 0.085 | 0.065** |  |  |  |
| MQ | 0.100*** | -0.001 | 0.067*** | 0.016 | 0.030 | 0.084*** | 0.035* |  |  |
| QTX | 0.175** | 0.092* | 0.110* | 0.064* | 0.011 | 0.094* | 0.078 | 0.085* |  |

*P<0.05; **P<0.02; ***P<0.001.

**Table E. Migration parameter (mean *M* and *θ* values) estimates for nine populations of *Lygus pratensis* based on the rDNA dataset.**

| Population |  | AKS | KT | WJQ | SC | SHZ | KC | KEL | MQ | QTX |
| --- | --- | --- | --- | --- | --- | --- | --- | --- | --- | --- |
| *i* | *θi* | →*i* | →*i* | →*i* | →*i* | →*i* | →*i* | →*i* | →*i* | →*i* |
| AKS | 0.00041 |  | 478.1 | 434.7 | 377.7 | 368.4 | 410.5 | 604.3 | 382.4 | 377.6 |
| KT | 0.00781 | 719.3 |  | 510.3 | 446.5 | 465.7 | 437.6 | 664.4 | 445.4 | 476.4 |
| WJQ | 0.01829 | 761.6 | 584.5 |  | 489.2 | 533 | 457.7 | 746.5 | 495.1 | 507.2 |
| SC | 0.06818 | 589.0 | 603.2 | 571.7 |  | 486.6 | 456.8 | 594.6 | 491.1 | 515.3 |
| SHZ | 0.05309 | 604.7 | 562.5 | 559.8 | 504.7 |  | 520.1 | 606.8 | 496.6 | 513.3 |
| KC | 0.00822 | 666.6 | 496.3 | 488.9 | 430.7 | 477.7 |  | 649.7 | 437.1 | 450.3 |
| KEL | 0.00055 | 611.9 | 479.5 | 450.9 | 396.7 | 432.5 | 561.3 |  | 428.0 | 422.4 |
| MQ | 0.03334 | 677.3 | 628.5 | 501.3 | 445.0 | 444.4 | 433.9 | 711.8 |  | 451.6 |
| QTX | 0.05805 | 637.0 | 542.2 | 519.9 | 493.2 | 485.0 | 493.4 | 649.1 | 518.3 |  |

Note: The ‘***i***’ indicates the population code in the first column; ‘***θ***’ indicates the mutation-scaled population size; ‘*M*’ indicates mutation-scaled migration rate.

**Table F. Number of effective migrants per generation (*Nem*) for nine populations of *Lygus pratensis* based on the mtDNA dataset.**

| Population |  | AKS | KT | WJQ | SC | SHZ | KC | KEL | MQ | QTX |
| --- | --- | --- | --- | --- | --- | --- | --- | --- | --- | --- |
| *i* | *θi* | →*i* | →*i* | →*i* | →*i* | →*i* | →*i* | →*i* | →*i* | →*i* |
| AKS | 0.01599 |  | 9.33 | 9.58 | 11.87 | 9.61 | 9.51 | 10.53 | 10.33 | 8.22 |
|  |  |  | 1.52-15.99 | **1.46-15.99** | **4.39-15.99** | 1.52-15.99 | 1.45-15.99 | **2.12-15.99** | **1.85-15.99** | 3.21-15.84 |
| KT | 0.00256 | 1.36 |  | 1.55 | 1.76 | 1.69 | 1.46 | 1.47 | 1.44 | 1.24 |
|  |  | 0.14-2.54 |  | 0.23-2.56 | **0.49-2.56** | 0.34-2.56 | 0.20-2.56 | 0.25-2.56 | 0.18-2.56 | 0.00-2.41 |
| WJQ | 0.00157 | 0.79 | 0.83 |  | 1.04 | 1.00 | 0.82 | 0.88 | 0.83 | 0.73 |
|  |  | **0.00-0.04** | 0.13-1.56 |  | 0.22-1.57 | 0.21-1.57 | 0.33-1.56 | 0.18-1.57 | 0.08-1.56 | **0.00-1.47** |
| SC | 0.00038 | 0.17 | 0.19 | 0.20 |  | 0.21 | 0.19 | 0.19 | 0.19 | 0.16 |
|  |  | **0-0.35** | **0.03-0.38** | 0.02-0.38 |  | **0.05-0.38** | **0.00-0.36** | 0.13-0.38 | 0.00-0.23 | **0.00-0.35** |
| SHZ | 0.01203 | 5.79 | 6.37 | 6.87 | 7.04 |  | 5.99 | 6.18 | 6.19 | 6.06 |
|  |  | 0.00-11.11 | 2.19-11.97 | 0.95-12.03 | **0.96-12.03** |  | 0.00-5.27 | **5.21-11.89** | **2.25-11.89** | 1.31-11.85 |
| KC | 0.01317 | 7.32 | 7.34 | 8.19 | 9.37 | 8.28 |  | 8.21 | 7.66 | 6.43 |
|  |  | 1.73-13.16 | 1.06-13.17 | 1.33-13.17 | **2.74-13.17** | 1.28-13.17 |  | **1.50-13.17** | **1.24-13.17** | 0.00-6.98 |
| KEL | 0.00091 | 0.51 | 0.45 | 0.50 | 0.57 | 0.54 | 0.47 |  | 0.49 | 0.46 |
|  |  | **0.07-0.91** | 0.00-0.66 | 0.08-0.91 | 0.11-0.91 | **0.09-0.91** | **0.05-0.90** |  | 0.06-0.91 | **0.04-0.90** |
| MQ | 0.00093 | 0.47 | 0.46 | 0.45 | 0.55 | 0.52 | 0.42 | 0.47 |  | 0.45 |
|  |  | **0.06-0.92** | 0.00-0.67 | 0.00-0.84 | 0.08-0.93 | **0.08-0.93** | **0-0.87** | 0.00-0.33 |  | **0.00-0.79** |
| QTX | 0.02193 | 11.84 | 12.11 | 12.77 | 14.10 | 13.97 | 11.83 | 12.90 | 15.86 |  |
|  |  | 2.05-21.86 | 1.58-21.91 | **1.90-21.93** | **2.81-21.93** | 2.43-21.93 | 1.46-21.87 | **1.89-21.93** | **5.23-21.93** |  |

Note: The black fonts indicate values of asymmetric migrants; the lines above indicate mean *Nem* value, the lines below indicate 97.5% highest probability distribution (HPD); ‘***i***’ indicates the population code in the first column; ‘***θ***’ indicates the mutation-scaled population size; ‘*M*’ indicates mutation-scaled migration rate.

**Table G. Number of effective migrants per generation (*Nem*) for nine populations of *Lygus pratensis* based on the rDNA dataset.**

| Population |  | AKS | KT | WJQ | SC | SHZ | KC | KEL | MQ | QTX |
| --- | --- | --- | --- | --- | --- | --- | --- | --- | --- | --- |
| *i* | *θi* | →*i* | →*i* | →*i* | →*i* | →*i* | →*i* | →*i* | →*i* | →*i* |
| AKS | 0.00041 |  | 0.20 | 0.18 | 0.15 | 0.15 | 0.17 | 0.25 | 0.16 | 0.15 |
|  |  |  | **0.00-0.38** | **0.00-0.37** | **0.00-0.36** | **0.00-0.36** | **0.00-0.37** | 0.04-0.41 | **0-0.37** | **0.00-0.37** |
| KT | 0.00781 | 5.62 |  | 3.99 | 3.49 | 3.64 | 3.42 | 5.19 | 3.48 | 3.72 |
|  |  | **1.94-7.81** |  | 0.36-7.69 | **0.00-7.1** | 0.00-6.51 | 0.00-7.22 | **1.26-7.81** | 0.00-7.21 | 0.00-6.84 |
| WJQ | 0.01829 | 13.93 | 10.69 |  | 8.95 | 9.75 | 8.37 | 13.65 | 9.06 | 9.28 |
|  |  | **5.21-18.29** | 1.45-18.29 |  | 0.00-16.67 | 1.05-18.19 | 0.00-15.42 | **4.40-18.29** | 0.00-14.76 | 0.87-18.08 |
| SC | 0.06818 | 40.16 | 41.13 | 38.98 |  | 33.18 | 31.14 | 40.54 | 33.48 | 35.13 |
|  |  | **6.59-68.18** | **7.96-68.18** | 5.86-68.18 |  | 0.00-63.05 | 0.00-63.54 | **6.22-68.18** | 0.00-29.5 | 6.18-67.41 |
| SHZ | 0.05309 | 32.10 | 29.86 | 29.72 | 26.79 |  | 27.61 | 32.22 | 26.36 | 27.25 |
|  |  | **5.27-53.09** | 3.79-53.05 | 4.18-53.02 | 2.34-52.45 |  | 2.59-52.56 | **5.13-53.09** | 0.42-31.85 | 6.69-52.49 |
| KC | 0.00822 | 5.48 | 4.08 | 4.02 | 3.54 | 3.93 |  | 5.34 | 3.59 | 3.70 |
|  |  | **1.45-8.22** | 0.22-6.89 | 0.00-7.34 | 0.00-7.38 | 0.00-7.43 |  | **1.18-8.22** | 0.00-7.55 | 0.00-7.65 |
| KEL | 0.00055 | 0.34 | 0.26 | 0.25 | 0.22 | 0.24 | 0.31 |  | 0.24 | 0.23 |
|  |  | 0.06-0.55 | **0.00-0.44** | **0.00-0.51** | **0.00-0.5** | **0.00-0.51** | **0.06-0.55** |  | **0-0.5** | **0.00-0.5** |
| MQ | 0.03334 | 22.58 | 20.95 | 16.71 | 14.84 | 14.82 | 14.47 | 23.73 |  | 15.06 |
|  |  | **5.27-33.34** | 7.78-33.34 | 0.00-12.16 | 0.00-29.43 | 0.00-30.81 | 0.00-30.76 | **7.89-33.34** |  | 0.00-31.03 |
| QTX | 0.05805 | 36.98 | 31.47 | 30.18 | 28.63 | 28.15 | 28.64 | 37.68 | 30.09 |  |
|  |  | **7.01-58.05** | 3.99-57.82 | 2.98-57.7 | 0.00-46.36 | 0.00-47.68 | 0.00-38.97 | **8.52-58.05** | 3.83-57.63 |  |

Note: The black fonts indicate values of asymmetric migrants; the lines above indicate mean *Nem* value, the lines below indicate 97.5% highest probability distribution (HPD); ‘***i***’ indicates the population code in the first column; ‘***θ***’ indicates the mutation-scaled population size; ‘*M*’ indicates mutation-scaled migration rate.

**Table H. Migration parameter (mean *M* and *θ* values) estimates for nine populations of *Lygus pratensis* based on the combined mtDNA and rDNA datasets.**

| Population |  | AKS | KT | WJQ | SC | SHZ | KC | KEL | MQ | QTX |
| --- | --- | --- | --- | --- | --- | --- | --- | --- | --- | --- |
| *i* | *θi* | →*i* | →*i* | →*i* | →*i* | →*i* | →*i* | →*i* | →*i* | →*i* |
| AKS | 0.00257 |  | 714.1 | 516.2 | 525.7 | 450.1 | 596.5 | 524.4 | 677.8 | 556.7 |
| KT | 0.00860 | 617.3 |  | 651.4 | 624.3 | 538.0 | 364.8 | 524.0 | 589.1 | 464.6 |
| WJQ | 0.00328 | 599.2 | 650.3 |  | 441.0 | 261.9 | 533.4 | 713.9 | 539.1 | 516.6 |
| SC | 0.04226 | 617.9 | 626.0 | 672.3 |  | 308.8 | 348.0 | 604.6 | 698.1 | 553.0 |
| SHZ | 0.03064 | 615.0 | 567.8 | 462.7 | 581.2 |  | 420.5 | 630.7 | 434.0 | 497.2 |
| KC | 0.00879 | 553.2 | 721.6 | 560.5 | 537.0 | 307.9 |  | 667.7 | 491.2 | 493.5 |
| KEL | 0.00217 | 556.8 | 627.6 | 636.0 | 624.3 | 346.0 | 614.4 |  | 558.3 | 629.7 |
| MQ | 0.00706 | 634.4 | 500.0 | 578.9 | 710.5 | 504.9 | 314.7 | 567.5 |  | 488.8 |
| QTX | 0.00603 | 596.7 | 385.8 | 422.1 | 631.6 | 415.1 | 482.9 | 608.9 | 507.1 |  |

Note: The ‘***i***’ indicates the population code in the first column; ‘***θ***’ indicates the mutation-scaled population size; ‘*M*’ indicates mutation-scaled migration rate.

**Table I. Number of effective migrants per generation (*Nem*) for nine populations of *Lygus pratensis* based on the combined mtDNA and rDNA datasets.**

| Population |  | AKS | KT | WJQ | SC | SHZ | KC | KEL | MQ | QTX |
| --- | --- | --- | --- | --- | --- | --- | --- | --- | --- | --- |
| *i* | *θi* | →*i* | →*i* | →*i* | →*i* | →*i* | →*i* | →*i* | →*i* | →*i* |
| AKS | 0.00257 |  | 0.94-2.57 | 1.37-2.57 | **1.69-2.57** | 0.13-1.56 | 0.37-2.57 | 0.53-2.55 | 0.68-2.57 | 0.36-2.57 |
| KT | 0.0086 | 1.94-8.57 |  | 2.26-8.6 | **3.21-8.6** | **0.36-2.65** | 0.00-6.89 | 0.00-0.96 | 2.10-8.6 | 0.38-3.36 |
| WJQ | 0.00328 | 1.57-3.28 | 1.29-3.28 |  | 0.42-2.36 | 0.00-1.96 | 0.42-2.68 | 1.15-3.28 | 0.00-1.09 | 0.37-2.41 |
| SC | 0.04226 | **6.90-42.26** | **10.14-42.26** | 11.19-42.26 |  | 0.00-36.09 | 0-29.61 | 9.92-42.26 | 16.40-42.26 | 0.00-12.99 |
| SHZ | 0.03064 | 0.00-2.27 | **5.68-30.43** | 0.00-3.53 | 11.30-30.64 |  | 0-27.64 | **9.83-30.64** | 0.00-27.39 | **2.39-19.45** |
| KC | 0.00879 | 1.55-8.74 | 3.90-8.79 | 1.85-8.64 | 0.71-8.53 | 0.00-6.21 |  | **3.46-8.79** | 0.83-8.44 | 6.57-8.79 |
| KEL | 0.00217 | 0.30-2.15 | 0.50-2.17 | 0.60-2.17 | 0.70-2.17 | **0.00-1.74** | **0.43-2.17** |  | **0.64-2.16** | 0.46-2.17 |
| MQ | 0.00706 | 1.57-7.06 | 0.53-3.67 | 1.07-7.02 | 2.48-7.06 | 0.00-1.09 | 0.38-4.48 | **4.25-7.06** |  | 0.07-3.32 |
| QTX | 0.00603 | 1.17-6.03 | 0.06-4.88 | 0.12-3.18 | 1.72-6.03 | **0.00-1.89** | 0.24-5.62 | 0.84-6.03 | 0.00-1.30 |  |

Note: The black fonts indicate values of asymmetric migrants; the lines above indicate mean *Nem* value, the lines below indicate 97.5% highest probability distribution (HPD); ‘***i***’ indicates the population code in the first column; ‘***θ***’ indicates the mutation-scaled population size; ‘*M*’ indicates mutation-scaled migration rate.
